# The Course of Minipuberty in Daughters of Women with Low Gestational Vitamin D Status

**DOI:** 10.3390/nu16142362

**Published:** 2024-07-21

**Authors:** Karolina Kowalcze, Robert Krysiak, Giuseppe Gullo, Johannes Ott

**Affiliations:** 1 Department of Patophysiology, Faculty of Medicine, Academy of Silesia, Rolna 43, 40-555 Katowice, Poland; 2 Department of Pediatrics in Bytom, Faculty of Health Sciences in Katowice, Medical University of Silesia, Stefana Batorego 15, 41-902 Bytom, Poland; 3 Department of Internal Medicine and Clinical Pharmacology, Medical University of Silesia, Medyków 18, 40-752 Katowice, Poland; rkrysiak@sum.edu.pl; 4 Department of Obstetrics and Gynecology, Villa Sofia Cervello Hospital, University of Palermo, 90146 Palermo, Italy; gullogiuseppe@libero.it; 5 Clinical Division of Gynecologic Endocrinology and Reproductive Medicine, Department of Obstetrics and Gynecology, Medical University of Vienna, 1090 Vienna, Austria; johannes.ott@meduniwien.ac.at

**Keywords:** females, reproductive organs, hypothalamic–pituitary–gonadal axis, pregnancy complications, saliva, urine, vitamin D status

## Abstract

Minipuberty is a term describing temporary, sex-specific activation of the hypothalamic–pituitary–gonadal axis, which is implicated in the development of male and female genital organs. Sons of women with low vitamin D during gestation were found to be characterized by increased activity of the reproductive axis and faster postnatal growth of genital organs. The current study assesses for the first time the course of minipuberty in female descendants of women with a vitamin D deficit. The study population included three matched groups of infant girls: children born to women with vitamin D deficiency (25OHD concentration below 50 nmol/L), offspring of women with vitamin D insufficiency (25OHD concentration between 50 and 75 nmol/L), and daughters of healthy females (25OHD concentration between 75 and 150 nmol/L). Salivary concentrations of estradiol, progesterone, 17-hydroxyprogesterone and androgens, as well as urine concentrations of FSH and LH, were assayed during the first 18 months of life (once a month in the first 6 months, bimonthly between months 6 and 12, and then every three months). At each visit, beyond hormone measurements, the investigators assessed the size of reproductive organs: ovaries, uterus and breasts. In daughters of mothers with vitamin D deficiency, concentrations of FSH, LH and estradiol were higher and detectable for a longer period of time, while ovarian volume, uterine length and breast diameter were greater than in the remaining groups. Children born to women with vitamin D insufficiency were characterized by higher FSH levels than daughters of healthy females, though the detection period was the same, and both groups differed in breast diameter. These findings suggest that low vitamin D status during gestation leads to more pronounced and longer-lasting activation of the reproductive axis, and is associated with increased dimensions of sexual organs, the magnitude of which depends on the degree of vitamin D deficit.

## 1. Introduction

Minipuberty is a term describing describing temporary, sex-specific activation of the hypothalamic–pituitary–gonadal axis, which occurs soon after birth [1,2,3,4,5]. It is chronologically the second activation of this axis, following the first phase taking place in the late first and the second trimester of pregnancy, and preceding the stage of physical maturation (puberty) that makes individuals capable of sexual reproduction [2]. Minipuberty in girls has a biphasic course, with an initial peak in serum concentrations of follicle-stimulating hormone (FSH), luteinizing hormone (LH) and estradiol at around days 15 to 27, and a second peak (less expressed than the early peak in case of gonadotropins) at around weeks 15 to 24 [6]. Infant girls are characterized by higher peak concentrations of FSH than LH (unlike the dominance of LH over FSH in boys), as well as by lower testosterone and higher estradiol concentrations than in boys [2,3]. Although LH levels in healthy girls decline at the age of around six months, FSH and estradiol concentrations may remain detectable up to the age of 3–4 years and up to the second year of life, respectively [3]. A physiological role of female minipuberty is still a matter of dispute. Minipuberty is thought to prime ovarian, uterine and breast tissues for subsequent development and maturation [4]. Moreover, early sex-specific activation of the reproductive axis is assumed to contribute to gender-related differences in growth velocity and fat distribution [5]. Lastly, minipuberty is speculated to regulate neuroglial plasticity, likely influencing sensory and cognitive maturation [7].

Numerous studies indicate that vitamin D (calciferol) is implicated in the development of the reproductive axis and physical maturation. A meta-analysis of six studies enrolling 3016 patients with precocious puberty and 8296 healthy controls showed that children with vitamin D deficiency were more prone to the development of precocious puberty, as compared with their peers with normal vitamin D status [8]. Another Chinese meta-analysis, including 2145 cases and 2063 healthy children participating in 15 studies (the majority of whom were girls), found an inverse correlation between 25-hydroxyvitamin D (25OHD) levels and central precocious puberty, and that vitamin D deficiency increased the risk of precocious puberty by 53.1% compared with healthy children [9]. In a meta-analysis by Wu et al., the odds ratio for precocious puberty in patients with low vitamin D status was equal to 2.25 [10]. Low serum 25OHD concentrations were found to increase the risk of idiopathic central precocious puberty independently of other risk factors [11]. Uterine volume in girls with idiopathic precocious puberty is inversely correlated with 25OHD concentrations [12]. Menarche occurred at an earlier age in girls with vitamin D deficiency than in girls with normal vitamin D status [13]. Girls diagnosed with premature adrenarche (the early presence of secondary sexual hair) were characterized by lower levels of 25OHD than healthy girls [14]. Lastly, low 25OHD levels were universally associated with central precocious puberty in girls, which did not differ depending on its forms [15]. Unfortunately, except for one prospective study in which 25OHD levels were measured only at baseline (before puberty) [13], the remaining ones were cross-sectional or case-control studies or were meta-analyses based on such studies [8,9,10,11,12,14,15]. The association between vitamin D homeostasis and the course of puberty seems to be likely also in light of studies investigating vitamin D receptor function. Vitamin D receptor GG Apal single nucleotide polymorphism was diagnosed more frequently in girls with precocious puberty than in controls, and estradiol and total testosterone levels differed between its alleles [16]. Interestingly, pubertal timing in the Danish population was earlier if the first trimester of the mother’s pregnancy fell in the period between November and April [17]. This phenomenon may be explained by lower vitamin D production in this period of the year [18], suggesting an association between vitamin D status during pregnancy and pubertal development.

Recently, our research team demonstrated for the first time that low vitamin D status during gestation was associated with increased activation of the hypothalamic–pituitary–testicular axis and accelerated growth of male genital organs in the first year of life, correlating with the degree of vitamin D deficit [19]. Unfortunately, no previous study has determined the association between maternal vitamin D status in pregnancy and the course of minipuberty in their daughters. Thus, it seems reasonable to investigate the activity of the reproductive axis and the postnatal development of female reproductive organs during minipuberty of daughters born to women with low gestational vitamin D status, which was the aim of the current study.

## 2. Materials and Methods

This research was a prospective, matched, outpatient cohort study, performed in the period between May 2022 and May 2024. The study was carried out in the outpatient pediatric departments of the provincial specialist hospitals in Bytom and Częstochowa, tertiary teaching hospitals localized in the Upper Silesia province, the most urbanized area of Poland. Because of its nature, clinical trial registration was not required. The study protocol was approved by the institutional ethics committee (approval number: PCN/CBN/0052/KB1/17/22), and complied with the principles outlined in the Helsinki Declaration. Written informed consent was obtained from the legal proxies (parents or guardians) of all participants before the study commenced. The reporting of the current work is compliant with Strengthening the Reporting of Observational Studies in Epidemiology (STROBE) guidelines.

### 2.1. Participants

All studied subjects and their mothers were white Polish Caucasians, referred to the study centers by cooperating physicians specializing in neonatology or obstetrics. The research included a group of 90 girls born in the past month, chosen from 188 infant girls fulfilling the inclusion and exclusion criteria. The aim of this selection was to match the study groups for the mother’s age, body mass index (BMI) and gestational age of delivery. The matching procedure has been described elsewhere [19,20,21]. In short, groups were matched for maternal age, education, occupational activity, gestational age at delivery, and the number of previous deliveries using an algorithm based on the minimum Euclidean distance rule.

In accordance with national recommendations, since birth, all participants had been supplemented with 400 IU (10 µg) of calciferol, administered in the form of twist-off capsules. At the first visit, on the basis of gestational 25OHD levels, the girls were allocated into one of three groups. To be included, at least two measurements in at least two trimesters had to be performed, and their results had to be consistent. The first group enrolled daughters born to mothers with calciferol deficiency, the second group included female descendants of women with calciferol insufficiency, and the third group (the control group) encompassed daughters of women with adequate calciferol status. Vitamin D deficiency, vitamin D insufficiency and normal vitamin D status were defined as a 25OHD concentration less than 50 nmol/L (20 ng/mL), between 50 and 75 nmol/L (20 and 30 ng/mL), and between 75 and 150 nmol/L (30 and 60 ng/mL), respectively [22]. A preliminary calculation showed that the estimated sample size required for a given effect size (20% between-group difference in estradiol concentration), statistical power (80%) and significance level (5%) was 75 infants (25 children per group). Considering possible withdrawals, the number of children included in each group (*n* = 30) was greater by 25% than the calculated minimum sample size. Because of a significant variation across the seasons of the year [22], the study included similar numbers of children conceived between December and February, between March and May, between June and August, and between September and November (8, 7, 6 and 9 infants born to vitamin D-deficient mothers; 7, 8, 7 and 8 girls born to vitamin D-insufficient mothers; and 8, 6, 9 and 7 infants born to vitamin D-sufficient mothers).

Potential participants were excluded if maternal 25OHD levels during gestation (a) were measured only once, (b) exceeded 150 nmol/L (60 ng/mL), or (c) were inconclusive (e.g., one value suggestive of calciferol deficiency, with another one suggesting insulin insufficiency). They were also excluded in case of birth before a gestational age of 36 weeks, a history of birth asphyxia, major congenital anomalies, congenital infections, metabolic disorders, genetic or inherited disorders, other chronic diseases and any chronic pharmacotherapy (except for vitamin D preparations). Lastly, children were not considered for enrollment if their mothers had any chronic disease, were treated during gestation and lactation with any drug for a period exceeding 7 days (except for vitamin/micronutrient supplements for pregnant and breastfeeding women), had any complications requiring urgent hospital admission, or were addicted to drugs or alcohol.

### 2.2. Study Design

**Figure 1:**
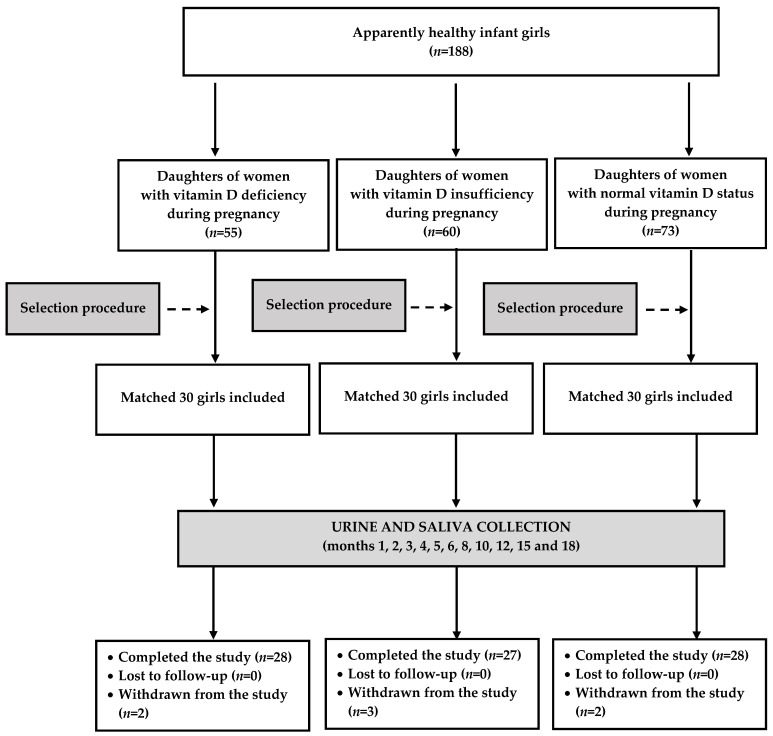
The flow of the participants through the study.

Figure 1 depicts the study flow from screening to the analysis. The participants underwent regular follow-up visits during the first 18 months of life: once a month during the first 6 months, bimonthly from months 7 to 12, and every three months from months 12 to 18. Throughout the study, the participants received the same dose of calciferol as prior to the study. At each visit, the parents or guardians were requested to describe the infant’s health status and they were questioned about the use of any prescription or non-prescription drugs or supplement medication since the previous visit. Additionally, the members of the research team performed a detailed clinical examination and interpreted imaging and laboratory findings. Saliva and urine were collected only if the child was healthy and treatment-naïve at the time of the study visit. Apart from exogenous calciferol, the exceptions were obligatory vaccinations and short-term treatment (no longer than a week), but only if there was a 10-day interval between treatment termination and the follow-up visit. The results of the participants were analyzed only if saliva and urine specimens were collected at least 8 times (on at least 5 occasions between months 1 and 6, at least 2 occasions between months 6 and 12, and at least one occasion between months 12 and 18). Adherence to vitamin D treatment was monitored at each visit by capsule counts, and was considered satisfactory if the percentage of unused capsules was below 10%. Total daily vitamin D intake was calculated at months 1, 3, 6, 12 and 18 by summing vitamin D intake from different food sources (milk, milk formulas, other foods) and from supplemental capsules. Vitamin D content in breast milk was calculated indirectly on the basis of total human milk intake and human milk vitamin D concentration, which depended on the infant’s age, the season and vitamin D supplementation by mothers. The necessary data were obtained from a meta-analysis of 43 studies [23]. For infants consuming only infant formula, daily vitamin D intake was determined based on the vitamin D content in the formula and the amount of formula. For infants consuming a mix of breast milk and infant formula, these intakes were added up. In older infants, vitamin D intake with food also included calciferol contained in dairy products, meat products, fish, eggs, cereal products, fruit and vegetables, and was calculated by analyzing 3-day food records.

At each visit, crown–heel length was measured to the nearest 1 mm from the top of the head to the sole of the foot with the infant lying supine using a mobile infantometer (Seca, Hamburg, Germany). Head circumference was measured with 1 mm precision with a tape on a line passing over the glabella and posterior occipital protrusion. Weight was measured to the nearest 10 g using a digital infant scale (Seca 834, Hamburg, Germany). The children were weighed without clothing and diapers. Body mass index (BMI) was calculated by dividing weight in kilograms by length in meters squared. Then, ultrasound imaging was performed using a high-resolution transducer (Esaote MyLab Six, Genoa, Italy), operating at frequencies ranging from 5 MHz to 12 MHz. Each measurement was repeated three times, and the mean value was used in further analyses. Ovarian volume was calculated using the prolate ellipsoid formula: 0.52 × length × depth × width. Length and depth were determined on parasagittal sonograms, while width was measured in transverse images. Mean ovarian volume was calculated by averaging the bilateral ovarian volume when both right and left ovaries could be visualized by ultrasound. When only one ovary could be measured, ovarian volume from this one ovary was used in the analysis. The uterine length was measured in the midsagittal plane from the fundus to the internal cervical os. Breast tissue size was measured to the nearest millimeter using a caliper, as described by Henriksen et al. [24]. Because values less than 3 mm are equivalent to the nipple diameter, they were regarded as unmeasurable and registered as 1 mm.

### 2.3. Laboratory Assays

All samples were collected in a quiet and temperature-controlled room between 7.30 and 9.00 a.m. Urine samples were obtained using collection bags (Medicavera, Szczecin, Polska). After cleaning and wiping the privates, the ends of the bag were attached on the skin above the privates and in the back of the vagina, with the sides next to each leg. Just after voiding, the bag was removed, and its content was poured into sterile tubes. Saliva specimens were collected by vacuum aspiration of 1 mL of saliva from the floor of the mouth. The whole procedure lasted from 25 to 50 s, was well tolerated, and was not stressful to the infants. In order to minimize the risk of contamination and potential interactions between feeding and salivary steroid hormones, saliva collection was performed not sooner than 60 min after the last feeding.

All assays were performed in duplicate to ensure consistency and precision. Urine levels of FSH and LH were assayed by chemiluminescent immunometric assay, and the obtained results were corrected for creatinine concentration (determined using a modified Jaffe method) to account for variable dilution among the samples [25]. In turn, salivary concentrations of steroids, including estradiol, testosterone, androstenedione, dehydroepiandrosterone sulfate (DHEA-S), progesterone and 17-hydroxyprogesterone were analyzed using an enzyme-linked immuno-sorbent assay [19,26]. Limits of detection (LODs) for the assayed hormones were as follows: 4 pmol/L for estradiol, 10 pmol/L for testosterone, 18 pmol/L for androstenedione, 100 nmol/L for DHEA-S, 16 pmol/L for progesterone, 11 pmol/L for 17-hydroxyprogesterone, 0.1 U/L for FSH and 0.1 U/L for LH.

### 2.4. Statistical Analysis

The LOD value was assigned for results below the LOD in statistical analyses if other results exceeded the LOD. All quantitative data underwent a logarithmic transformation to normalize the distribution prior to analysis. The study groups were compared by analysis of variance, with post hoc analysis performed using Bonferroni’s test. Categorical variables were compared using the chi-square test. The dynamics of hormone concentrations and organ sizes over time were modelled using linear mixed effect models, the strength of which accommodates missing data points, and they do not require the same number of observations per subject. Age at each visit and baseline values were included as fixed factors in the models, and subjects were included as a random factor. Age was treated as a categorical variable. Relationships between the assessed variables were explored by computing bivariate correlations using Pearson’s r tests. The significance threshold was set at a *p* value adjusted for multiple comparisons equal to 0.05. All statistical analyses were performed using the Statistica 12.0 PL software package (number: JPZP507D199115ARCN-E, StatSoft Polska, Kraków, Poland).

## 3. Results

Eighty-three of ninety included children (92.2%) completed the follow-up, and their results were statistically analyzed. Two girls and one infant, born, respectively, to mothers with vitamin D insufficiency and deficiency, dropped out because of recurrent acute infections that made it impossible to collect the required number of urine and saliva samples. Another two children (of calciferol-sufficient mothers) did not complete the study because they were diagnosed during infancy with food allergy and asthma, requiring chronic therapy. One infant (born to a mother with vitamin D deficiency) dropped out after being informed about preeclampsia in her mother (not mentioned during recruitment). Lastly, the parents of one infant (of a mother with calciferol insufficiency) withdrew their daughter from the study without giving a reason. There were no cases of poor adherence to vitamin D supplementation.

At baseline, there was no difference between the groups of infant girls in terms of gestational age of delivery, birth order, anthropometric variables (length, head circumference, weight and BMI) and total daily calciferol intake. The percentage of breastfed infants was similar in all study groups (Table 1). The studied groups also did not differ in total daily vitamin D intake at months 3, 6, 12 and 18 (Supplementary Table S1).

Age, education, employment, BMI and blood pressure did not differ between the mothers of the infant girls participating in the study. The percentage of women smoking during gestation was similar. However, the study groups differed in 25OHD concentrations, daily calciferol intake and cumulative calciferol intake during gestation. The lowest and the highest values were observed in vitamin D-deficient and vitamin D-sufficient mothers, respectively (Table 2).

**Figure 2:**
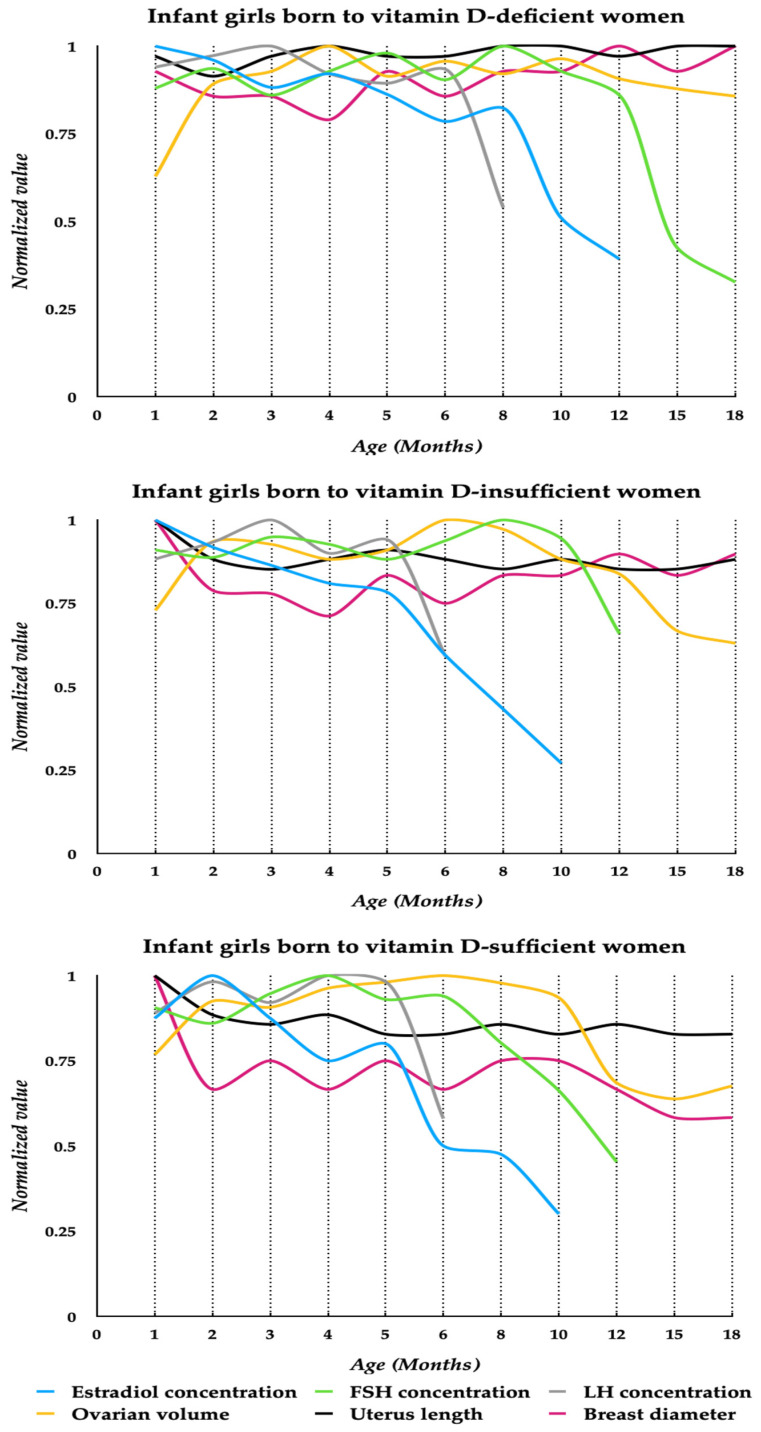
Average curves of reproductive hormones and organ sizes in infant girls during the first 18 months of life. Estradiol concentration, FSH concentration, LH concentration, ovarian volume, uterus length and breast diameter are expressed as normalized values (mean hormone concentration/mean organ size divided by their maximum value). Abbreviations: FSH—follicle-stimulating hormone; LH—luteinizing hormone.

Estradiol was detectable in saliva from month 1 to month 12 in the offspring of mothers with vitamin D deficiency, and from month 1 to month 10 in the infants born to vitamin D-insufficient and vitamin D-sufficient women. Significant decreases in estradiol concentrations were observed after month 8 in the infants born to calciferol-deficient mothers and after month 5 in the remaining groups (Figure 2). During the first 12 months of life, estradiol concentrations were higher in descendants of vitamin D-deficient mothers than in the remaining groups, but did not differ between infants born to calciferol-insufficient and calciferol-sufficient women (Table 3).

There were no between-group differences in saliva concentrations of testosterone (Supplementary Table S2), androstenedione (Supplementary Table S3), DHEA-S (Supplementary Table S4), progesterone (Supplementary Table S5) and 17-hydroxyprogesterone (Supplementary Table S6) at any time point. Testosterone and androstenedione levels exceeded the LOD until the age of 5 months, while the remaining steroids were detectable from month 1 to month 12. 

In daughters born to mothers with vitamin D deficiency, FSH was detectable in urine for the whole observation period, whereas this was the case in the remaining groups only from month 1 to month 12. Stable levels were found from month 1 to month 12 in infants of vitamin D-deficient women, from month 1 to month 10 in infants of vitamin D-insufficient women, and from month 1 to month 6 in infants of vitamin D-sufficient women, and decreased thereafter (Figure 2). For the entire observation period, urinary FSH levels were higher in daughters born to mothers with vitamin D deficiency than in the remaining groups, while between months 8 and 12, they were also higher in infants of women with vitamin D insufficiency than in infants of women with normal vitamin D status (Table 4). 

Urinary LH levels were detectable in the first 8 months of life in daughters of vitamin D-deficient women, and in the first 6 months of life in the remaining groups. The levels were stable between months 1 and 6 in infants born to calciferol-deficient women, or between months 1 and 5 in descendants of vitamin D-insufficient and vitamin D-sufficient women, and decreased thereafter (Figure 2). Over the entire period of detection, urinary LH concentrations were higher in the offspring of vitamin D-deficient women than in the remaining groups, but did not differ between daughters born to mothers with vitamin D insufficiency and with normal vitamin D status (Table 5).

In all study groups, ovarian volume increased from month 1 to month 2, and remained stable thereafter. In daughters born to mothers with vitamin D deficiency during pregnancy, ovarian volume remained stable throughout the study. In daughters born to mothers with vitamin D insufficiency, ovarian volume was greater between month 2 and month 12 than in months 15 and 18. In the offspring of women with normal calciferol homeostasis, this volume was stable between months 2 and 10, and decreased thereafter (Figure 2). Except for month 1, ovarian volume was greater in infants born to mothers with calciferol deficiency than in the remaining groups, but did not differ between daughters born to mothers with vitamin D insufficiency and with normal vitamin D status (Table 6).

In infants of women with calciferol deficiency during pregnancy, uterine length did not change throughout the study. In the remaining groups, uterine length was slightly but significantly shorter in month 2 than in month 1, remaining unchanged thereafter (Figure 2). From month 3 to month 18, the uterus was longer in descendants of calciferol-deficient women than in the offspring of women with vitamin D deficiency and normal vitamin D status. There were no differences in uterine length between infants born to mothers with vitamin D insufficiency and with normal vitamin D status (Table 7).

In infants born to women with vitamin D deficiency, there were no differences in breast diameter over the entire study period. In girls born to calciferol-insufficient mothers, the largest breast diameter was observed at month 1, but differences in this parameter at different time points did not reach statistical significance. In daughters of women with normal calciferol status during pregnancy, its size decreased between month 1 and month 2, with no changes thereafter (Figure 2). Except for month 1, breast diameter was higher in the offspring of women with gestation vitamin D deficiency than in the remaining groups. From month 12 to month 18, this diameter differed between daughters born to mothers with vitamin D insufficiency and with normal vitamin D status (Table 8).

Table 9 shows correlations between the assessed variables. Maternal 25OHD concentrations correlated with cumulative maternal vitamin D intake. Throughout the detection period, in all study groups, there were positive correlations between infants’ estradiol levels and LH concentrations, between ovarian volume and LH and FSH concentrations, between uterine length and estradiol levels, and between breast diameter and estradiol and FSH concentrations. In daughters born to vitamin D-deficient mothers, 25OHD concentrations inversely correlated with estradiol, LH and FSH levels. In infants of vitamin D-insufficient women, 25OHD concentrations inversely correlated only with FSH concentrations. Other correlations did not reach the level of statistical significance.

## 4. Discussion

The major finding of the current study is that long-lasting gestational vitamin D deficiency results in a stronger activation of the hypothalamic–pituitary–ovarian axis in the female offspring. Compared to infant girls born to healthy women with normal calciferol homeostasis, female descendants of mothers with 25OHD concentrations consistently below 50 nmol/L were characterized by higher levels of both gonadotropins and estradiol, and by longer periods of their detection. Positive correlations between urinary LH and salivary estradiol levels in all treatment groups indicate that overactivation of this axis is, at least in the first stage of minipuberty, a consequence of increased and prolonged activation of pituitary cells secreting gonadotropins (gonadotropes). The obtained results are generally similar to those reported recently in sons of women with different calciferol statuses during pregnancy [19]. This similarity suggests that the impact of impaired calciferol homeostasis on the course of minipuberty is sex-independent. An interesting difference between infant girls and infant boys, irrespective of whether they were born to women with hypovitaminosis D or to women with normal vitamin D status, was the lack of evident peak concentrations in the former. This dissimilarity probably reflects fluctuations in the secretion of estradiol (and maybe also of gonadotropins) in response to cyclic folliculogenesis and follicular atrophy [27], which in individual patients are temporarily uncoordinated. Because of very rigorous inclusion criteria, the study findings do not seem to result from the impact of concurrent pathological conditions or treatments in both mothers and daughters.

There are very few repeated measures data on organ size and hormone concentrations during female minipuberty. The sizes of the uterus and breasts in daughters born to vitamin D-sufficient women were similar to those observed by Kuiri-Hänninen et al. [27], and like these authors, we observed an early decrease in their size. In turn, ovarian volume was only slightly greater than reported in a longitudinal study by Chin et al. [28], who also found slight ovarian enlargement in the first weeks after birth. Thus, changes in the ovaries, uterus and breasts in the first 18 months of life do not seem to be determined by ethnic and socioeconomic considerations. Because of the paucity of studies, a more difficult task is to compare hormone concentrations. However, our findings concerning urinary gonadotropin levels in female infants of vitamin D-sufficient women resemble those of Kuiri-Hänninen et al. [29,30], though the FSH concentrations we found were slightly higher but detectable for a shorter period of time, while the LH levels we found were greater than in their study. In turn, the salivary estradiol concentrations we found were higher than those reported in the only study assessing salivary estradiol levels in infant girls [31]. Because of the same assay method, the observed differences cannot be explained by overestimation of salivary estradiol concentration, but seem to result from differences in the studied populations, inclusion criteria, and the number of estradiol measurements (only a single measurement in the study by Kalaycı et al.) [31].

As expected, estradiol levels were higher in the participants of the present study than in the boys participating in the previous one [19]. More importantly, irrespective of the study group, the detection period was longer for estradiol than for testosterone. These findings are in line with a previous study that showed that gonads isolated from girls, who had died suddenly between birth and two years of age, contained higher amounts of estradiol and estrone than the gonads of peer boys did [32]. Positive correlations between estradiol levels and ovarian volume, and between estradiol and LH concentrations, indirectly argue that raised estradiol concentrations in daughters of women with gestational calciferol deficiency result from increased ovarian production of this hormone, induced by gonadotroph overactivity. Despite the physiologically low activity in childhood [33], differences in the detection periods between estradiol and androgens (testosterone and androstenedione), and the lack of correlations between salivary levels of estradiol and androgens, we cannot fully exclude the additional role of enhanced extra-ovarian aromatase activity. In line with this interpretation, calcitriol was found to inhibit the activity of this enzyme in extra-gonadal tissues (though obtained from adult subjects) [34,35]. Thus, low availability of the upstream substrate for calcitriol production may putatively activate aromatase activity and extra-ovarian estradiol production.

The raised activity of the hypothalamic–pituitary–ovarian axis in children of vitamin D-deficient women does not seem to be a consequence of differences in postnatal calciferol homeostasis in the participating infants. The total intake of this vitamin was similar in all study groups, and over the entire study period, all girls received additional vitamin D supplementation at the same daily dose of 10 µg, which is considered enough to normalize calciferol status [22]. Our findings suggest, rather, that the altered course of minipuberty in daughters born to women with hypovitaminosis D reflects chronically low availability of this vitamin during pregnancy, and cannot be explained by pharmacokinetic and/or pharmacodynamic interactions of exogenous calciferol preparations at the level of the hypothalamic–pituitary–ovarian axis. In line with these explanations, concentrations of hormones of the reproductive axis correlated with 25OD levels but not with calciferol intake in women, though there were marked between-group differences in daily vitamin D intake and cumulative vitamin D intake by mothers during pregnancy. 

Very limited knowledge on the physiological role of the first two activations of the hypothalamic–pituitary–gonadal axis (in utero and during infancy) and the chosen study design make it impossible to identify mechanisms explaining the association between hypovitaminosis D and overactivity of the reproductive axis in the first postnatal months. Another hindering factor is the lack of data concerning the course of female minipuberty in other metabolic and endocrine disorders. Overactivity of the reproductive axis in daughters of calciferol-deficient women may represent a specific response to disturbances in calciferol homeostasis, but it is also likely that the developing organism responds in the same way to different factors disturbing the intrauterine environment. Inverse correlations between salivary concentrations of estradiol and urinary levels of gonadotropins and maternal 25OD concentrations support the first mechanism. It should be underlined that most measurements of 25OHD were performed in the late first trimester and in the second trimester, temporarily corresponding to the period of the first (intrauterine) phase of gonadal axis activation [2]. Low vitamin D status in this period of gestation may affect intrauterine production of gonadotropins and estrogens, with resultant changes at the level of the reproductive axis itself and/or target organs, both of which may modulate the course of the second phase (minipuberty). Some evidence suggests a possible involvement of 5′-adenosine monophosphate-activated protein kinase. Reduced activity of 5′-adenosine monophosphate-activated protein kinase in neurons secreting gonadotropin-releasing hormone is implicated in the induction and progress of puberty [36,37]. Moreover, vitamin D seems to modulate gonadotropin secretion in adult women by affecting hypothalamic and/or pituitary production of this kinase [38]. According to the alternative explanation, maternal hypovitaminosis D may be a factor impairing programming during embryonic and fetal life, which is a process determining the set point for various physiological responses, including those important for postnatal sexual maturation [39]. 

The obtained results allow us to draw some conclusions about the clinical consequences of increased activation of the reproductive axis in the first months of life. Not only ovarian volume, but also uterine length and breast diameter were greater in daughters of women with vitamin D deficiency than in the remaining female descendants participating in the current study. Moreover, the size of these organs positively correlated with estradiol levels, while breast diameter additionally correlated with FSH concentrations. It should be underlined that between-group differences in the target organs reached statistical significance already in the second or third month of life, persisting to the end of the analyzed time period. Interestingly, there is a striking similarity between our findings and those reported by other authors [12], who observed that girls with idiopathic central precocious puberty displayed higher uterine volume if they were vitamin D deficient than in cases of vitamin D insufficiency or normal calciferol homeostasis. Thus, it is possible that larger dimensions of both sexual organs in the female offspring of women with gestational vitamin D deficiency may also be present in the postminipubertal period of physiological quiescence of the reproductive axis, and may potentially play a role in their final growth during puberty. Higher levels of estradiol and FSH already in the first month of life may explain the lack of an initial decrease in uterine and breast dimensions in daughters of vitamin D-deficient women, though such a decrease in both target organs was observed between months 1 and 2 in infants born to healthy women, and, even to a greater degree (probably because of a younger age at recruitment), between postnatal day 7 and postnatal months 2 and 3 in full-term infant girls born to healthy non-smoking Finnish women [27]. These differences may well be explained by the fact that higher concentrations of estradiol and gonadotropins in descendants of vitamin D-deficient women were observed already in the first month of life and were present over the entire observation period.

Although overactivity of the reproductive axis was more pronounced in cases of gestational vitamin D deficiency than gestational vitamin D insufficiency, the latter condition also seems to impact female minipuberty, though this effect is limited to more pronounced FSH secretion and breast growth. This finding is in line with our previous observations that calciferol insufficiency in mothers of boys is followed by an increase in postnatal FSH levels and testicular volume [19]. Interestingly, compared to children exposed in utero to normal gestational calciferol status, raised salivary FSH concentrations were evidenced later in girls (between months 8 and 12) than in boys (during the first 5 months of life). Although we cannot explain the reason for this discrepancy, the reason may be associated with a much longer detection period for FSH in infant girls than in infant boys [2]. Positive correlations between FSH and breast diameter suggest that higher production of this hormone may partially explain the greater diameter of the breasts from month 12 to month 18. This finding is in line with previously documented associations between breast growth and increased FSH production and between FSH predominance in children and the thelarche variant of gonadotropin-dependent precocious puberty (no extramammary signs of sexual maturation) [40]. It should be underlined that differences in breast diameter between daughters of vitamin D-insufficient and of healthy women were observed at the end of the study, but the clinical relevance of this finding is unclear, though it cannot be excluded that they are of some importance at later stages of sexual maturation [41,42]. Thus, this issue requires further investigation.

This study also provided other findings that may be clinically relevant. Firstly, salivary concentrations of steroid hormones in infant girls were detectable for a period no longer than 12 months. During this period, saliva may be the best biological material for evaluation of circulating steroids because of relatively high salivary concentrations, abundant production of saliva in the first year of life and an almost stress-free collection procedure. Secondly, maternal vitamin D status does not determine salivary concentrations of testosterone, androstenedione, DHEA-S, progesterone and 17-hydroxyprogesterone, at least during the first 18 months of life, arguing against the role of calciferol homeostasis during pregnancy in the regulation of androgen production in infant girls. No between-group differences in salivary testosterone are in contradiction with the results obtained by Kılınç et al. [43]. They reported higher serum total testosterone levels in infant girls aged from 30 to 45 days with serum 25OHD concentrations below 50 nmol/L than above this threshold. However, Kılınç et al. [43] did not investigate maternal health during gestation, the dynamic of testosterone concentration (measurements were performed only once for each participant), and they did not investigate the clinical significance of their findings by assessing target organs. Nevertheless, these inconclusive results suggest that the impact on female minipuberty may partially depend on the stage of life in which low vitamin D status occurs. Thirdly, different detection periods for individual steroids, as well as different concentrations compared with infant boys (even born to healthy mothers) [19,21] suggest that reference values for salivary steroids in infancy should be age- and gender-specific. Fourthly, the presence of 17-hydroxyprogesterone, progesterone and DHEA-S for the whole first year of life may help to diagnose non-classic congenital hyperplasia, which is characterized by their overproduction but remains often undiagnosed by neonatal screening (obligatory in most developed countries) [44]. Furthermore, assessment of these steroids in saliva may be useful in determining the appropriate glucocorticoid dosage for patients with classic forms of this disorder in the first postnatal year.

For ethical reasons (pain exposure), we did not assess 25OHD concentrations in the plasma/serum of infants participating in our study. We also did not measure 25-hydroxyvitamin D concentrations in saliva because of very low salivary levels, the existence of only a weak correlation between serum and salivary 25-hydroxyvitamin D, marked daily fluctuations in salivary 25-hydroxyvitamin D, dependence on the saliva flow rate, and no previous studies in children, all of which might have complicated sample collection protocols and measurements of 25-hydroxyvitamin D [45,46]. Instead, vitamin D intake in infants was calculated indirectly and, because of complexity, only at some visits (at months 1, 3, 6, 12 and 18). Despite the fact that there were no between-group differences in calciferol intake by infant girls participating in our study and excellent adherence, such a relationship cannot be totally excluded. There are also other study limitations that need to be acknowledged. Despite being adequately powered for the primary outcome, the small sample size limits the interpretation of the results. Because the present research was a cohort study, potential selection and confounding bias might have influenced the results. The relatively late time of the patients’ enrollment (the second half of the first month of life) excluded the possibility of assessing the earliest period of minipuberty (the first days after delivery). Because of cross-reactivity with steroid intermediates [47], immunoassays used in the current study might have overestimated salivary concentrations of the investigated steroids. The study design does not allow us to explain molecular and cellular mechanisms lying at the foundation of differences in the course of minipuberty between daughters born to healthy women and born to women with hypovitaminosis D. Lastly, despite taking many precautions in planning the study design and analyzing the obtained results, the potential influence of regression toward the mean cannot be completely ruled out [48].

## 5. Conclusions

Low gestational calciferol status affects hypothalamic–pituitary–gonadal axis activity in infant girls, and this impact correlates with the degree of hypovitaminosis D during pregnancy. Increased hormonal activity of gonadotropes, resulting in raised estradiol secretion, may partially explain different dimensions of female sexual organs (the ovaries, uterus and breasts) during the first 18 months of life. The presence of elevated FSH secretion and increased breast diameter in daughters of vitamin D-insufficient women suggest that even mild disturbances in calciferol homeostasis may influence the course of female minipuberty. Although long-term consequences of the altered course of minipuberty still require better understanding, both current findings and similar observations concerning male descendants suggest that hypovitaminosis D in pregnant women, irrespective of its severity, requires prompt diagnosis and treatment. We believe that our study will serve as a foundation for future larger-scale research, aimed at confirming the obtained novel results. It also seems interesting to investigate a relationship between other maternal micro- and macronutrient deficiencies in pregnancy and the course of minipuberty.

## Figures and Tables

**Table 1:** Baseline characteristics of infant girls by maternal vitamin D status during pregnancy.

Variable	Vitamin D Deficient	Vitamin D Insufficient	Vitamin D Sufficient	*p*-Values		
				Deficient vs. Insufficient	Deficient vs. Insufficient	Insufficient vs. Sufficient
Number (*n*)	28	27	28	-	-	-
Gestational age at delivery (weeks)	39 ± 2	39 ± 2	40 ± 1	1.0000	0.2430	0.2621
Birth order: first/second/third and subsequent (%)	50/43/7	44/44/11	46/43/11	0.7236	0.7545	0.8375
Body length (cm)	54.1 ± 1.8	53.8 ± 1.5	54.3 ± 1.9	0.5057	0.6875	0.2848
Head circumference (cm)	37.0 ± 0.8	37.2 ± 0.7	36.8 ± 0.8	0.3290	0.3537	0.0896
Body weight (kg)	4.28 ± 0.52	4.40 ± 0.53	4.43 ± 0.55	0.4006	0.2988	0.8380
BMI (kg/m^2^)	14.6 ± 1.4	15.2 ± 1.3	15.0 ± 1.1	0.1158	0.2398	0.5402
Breastfeeding (%)	79	85	82	-	-	-
Total daily vitamin D intake (µg)	13.1 ± 1.5	13.2 ± 1.7	13.4 ± 1.8	0.8178	0.5010	0.6738

The data are shown as the mean ± standard deviation (unless otherwise stated). Abbreviation: BMI—body mass index.

**Table 2:** Baseline characteristics of mothers of the infant girls who completed the study.

Variable	Vitamin D Deficient	Vitamin D Insufficient	Vitamin D Sufficient	*p*-Values		
				Deficient vs. Insufficient	Deficient vs. Insufficient	Insufficient vs. Sufficient
Number (*n*)	28	27	28	-	-	-
Age (years)	34 ± 9	34 ± 9	35 ± 8	1.0000	0.6792	0.6820
University/secondary/primary or vocational education (%)	39/43/18	44/41/15	43/43/14	0.6231	0.6084	0.7325
Employment rate/white-collar/pink-collar/blue-collar workers (%)	86/25/29/32	85/19/30/37	82/29/32/21	0.7568	0.8228	0.7611
Smoking during pregnancy (%)	32	30	29	-	-	-
BMI (kg/m^2^)	24.8 ± 4.1	24.1 ± 3.7	23.9 ± 3.8	0.5096	0.3980	0.8441
Systolic blood pressure (mmHg)	122 ± 20	118 ± 16	116 ± 18	0.4175	0.2432	0.6654
Diastolic blood pressure (mmHg)	81 ± 6	80 ± 6	79 ± 7	0.5394	0.2561	0.5725
Mean 25OHD concentrations (nmol/L)	34 ± 8	63 ± 7	114 ± 20	<0.0001	<0.0001	<0.0001
Mean daily vitamin D intake during pregnancy (µg)	11.2 ± 3.7	18.1 ± 4.3	41.2 ± 11.8	<0.0001	<0.0001	<0.0001
Cumulative vitamin D intake during pregnancy (mg)	3.9 ± 1.5	6.0 ± 1.8	13.1 ± 3.7	<0.0001	<0.0001	<0.0001

Unless otherwise stated, the data are shown as the mean ± standard deviation. BMI and blood pressure represent mean values from visits during which 25OHD concentrations were registered. Abbreviations: BMI—body mass index; 25OHD—25-hydroxyvitamin D.

**Table 3:** Estradiol concentrations in saliva of infant girls born to mothers with different vitamin D statuses during pregnancy.

Study Month	Vitamin D Deficient	Vitamin D Insufficient	Vitamin D Sufficient	*p*-Values		
				Deficient vs. Insufficient	Deficient vs. Insufficient	Insufficient vs. Sufficient
1	51 ± 20	37 ± 18	35 ± 16	0.0087	0.0017	0.6647
2	49 ± 16	34 ± 14	40 ± 12	0.0005	0.0208	0.0934
3	45 ± 17	32 ± 14	35 ± 14	0.0032	0.0197	0.3968
4	47 ± 19	30 ± 15	30 ± 18	0.0006	0.0011	1.0000
5	44 ± 20	29 ± 16	32 ± 19	0.0034	0.0253	0.6385
6	40 ± 23	22 ± 14	20 ± 12	0.0010	0.0001	0.5714
8	42 ± 18	16 ± 8	19 ± 14	<0.0001	<0.0001	0.3460
10	26 ± 10	10 ± 7	12 ± 7	<0.0001	<0.0001	0.2943
12	20 ± 12	Below LOD	Below LOD	<0.0001	<0.0001	-

The data are shown as the mean in (pmol/L) ± standard deviation. For between-group statistical comparisons, LOD value was assigned for estradiol in infants born to vitamin D-insufficient and vitamin D-sufficient women at month 12. Abbreviation: LOD—limit of detection.

**Table 4:** Urinary FSH levels in infant girls born to mothers with different vitamin D statuses during pregnancy.

Study Month	Vitamin D Deficient	Vitamin D Insufficient	Vitamin D Sufficient	*p*-Values		
				Deficient vs. Insufficient	Deficient vs. Insufficient	Insufficient vs. Sufficient
1	2.20 ± 0.81	1.63 ± 0.71	1.56 ± 0.80	0.0077	0.0044	0.7329
2	2.34 ± 0.75	1.59 ± 0.80	1.48 ± 0.75	0.0007	0.0001	0.6009
3	2.15 ± 0.68	1.70 ± 0.91	1.63 ± 0.59	0.0421	0.0035	0.7341
4	2.32 ± 0.80	1.66 ± 0.67	1.72 ± 0.64	0.0017	0.0031	0.7365
5	2.45 ± 0.90	1.58 ± 0.82	1.60 ± 0.73	0.0008	0.0002	0.9290
6	2.26 ± 0.83	1.68 ± 0.93	1.62 ± 0.80	0.0180	0.0049	0.7980
8	2.50 ± 1.01	1.79 ± 0.85	1.38 ± 0.60	0.0068	<0.0001	0.0431
10	2.32 ± 0.78	1.69 ± 0.64	1.14 ± 0.63	0.0019	<0.0001	0.002
12	2.15 ± 0.84	1.18 ± 0.55	0.78 ± 0.52	<0.0001	<0.0001	0.0077
15	1.06 ± 0.62	Below LOD	Below LOD	<0.0001	<0.0001	-
18	0.82 ± 0.55	Below LOD	Below LOD	<0.0001	<0.0001	-

The data are shown as the mean (in international units per mmol of creatinine) ± standard deviation. For between-group statistical comparisons, LOD value was assigned for FSH in infants born to vitamin D-insufficient and vitamin D-sufficient women at months 15 and 18. Abbreviations: FSH—follicle-stimulating hormone; LOD—limit of detection.

**Table 5:** Urinary LH levels in infant girls born to mothers with different vitamin D statuses during pregnancy.

Study Month	Vitamin D Deficient	Vitamin D Insufficient	Vitamin D Sufficient	*p*-Values		
				Deficient vs. Insufficient	Deficient vs. Insufficient	Insufficient vs. Sufficient
1	1.43 ± 0.60	1.06 ± 0.40	0.98 ± 0.44	0.0098	0.0023	0.4840
2	1.48 ± 0.53	1.12 ± 0.52	1.12 ± 0.55	0.0140	0.0157	1.0000
3	1.52 ± 0.55	1.20 ± 0.60	1.05 ± 0.60	0.0440	0.0035	0.3582
4	1.40 ± 0.48	1.08 ± 0.56	1.14 ± 0.62	0.0268	0.0450	0.7083
5	1.36 ± 0.51	1.13 ± 0.46	1.12 ± 0.49	0.0452	0.0443	0.9381
6	1.42 ± 0.50	0.71 ± 0.35	0.65 ± 0.32	<0.0001	<0.0001	0.5096
8	0.82 ± 0.46	Below LOD	Below LOD	<0.0001	<0.0001	-

The data are shown as the mean (in international units per mmol of creatinine) ± standard deviation. For between-group statistical comparisons, LOD value was assigned for LH in infants born to vitamin D-insufficient and vitamin D-sufficient women at month 8. Abbreviations: LH—luteinizing hormone; LOD—limit of detection.

**Table 6:** Ovarian volume in infant girls born to mothers with different vitamin D statuses during pregnancy.

Study Month	Vitamin D Deficient	Vitamin D Insufficient	Vitamin D Sufficient	*p*-Values		
				Deficient vs. Insufficient	Deficient vs. Insufficient	Insufficient vs. Sufficient
1	0.88 ± 0.30	0.81 ± 0.30	0.83 ± 0.28	0.3908	0.5218	0.7992
2	1.25 ± 0.35	1.04 ± 0.28	1.00 ± 0.24	0.0176	0.0029	0.5714
3	1.30 ± 0.46	1.03 ± 0.40	0.98 ± 0.25	0.0305	0.0024	0.6083
4	1.40 ± 0.52	0.98 ± 0.39	1.04 ± 0.36	0.0076	0.0018	0.5035
5	1.28 ± 0.38	1.01 ± 0.40	1.06 ± 0.40	0.0131	0.0395	0.6449
6	1.34 ± 0.43	1.11 ± 0.37	1.08 ± 0.42	0.0385	0.0260	0.7800
8	1.29 ± 0.35	1.08 ± 0.42	1.05 ± 0.40	0.0487	0.0204	0.7872
10	1.35 ± 0.48	0.98 ± 0.31	1.01 ± 0.30	0.0014	0.0025	0.7168
12	1.27 ± 0.41	0.93 ± 0.38	0.74 ± 0.32	0.0024	<0.0001	0.0688
15	1.23 ± 0.49	0.74 ± 0.28	0.69 ± 0.30	<0.0001	<0.0001	0.5259
18	1.20 ± 0.46	0.70 ± 0.28	0.73 ± 0.28	<0.0001	<0.0001	0.6928

The data are shown as the mean (in mL) ± standard deviation.

**Table 7:** Uterine length in infant girls born to mothers with different vitamin D statuses during pregnancy.

Study Month	Vitamin D Deficient	Vitamin D Insufficient	Vitamin D Sufficient	*p*-Values		
				Deficient vs. Insufficient	Deficient vs. Insufficient	Insufficient vs. Sufficient
1	34 ± 5	34 ± 6	35 ± 6	1.0000	0.5010	0.5393
2	32 ± 6	30 ± 5	31 ± 5	0.1858	0.4617	0.4617
3	34 ± 5	29 ± 6	30 ± 5	0.0014	0.0042	0.5042
4	35 ± 5	30 ± 5	31 ± 6	0.0005	0.0090	0.5057
5	34 ± 4	31 ± 5	29 ± 5	0.0171	0.0001	0.1440
6	34 ± 6	30 ± 5	29 ± 5	0.0018	0.0013	0.4617
8	35 ± 5	29 ± 4	30 ± 4	<0.0001	0.0001	0.3582
10	35 ± 4	30 ± 4	29 ± 4	0.0001	<0.0001	0.3582
12	34 ± 4	29 ± 5	30 ± 5	0.0001	0.0017	0.4617
15	35 ± 5	29 ± 4	29 ± 5	<0.0001	<0.0001	1.0000
18	35 ± 4	30 ± 5	29 ± 4	0.0001	<0.0001	0.4203

The data are shown as the mean (in mm) ± standard deviation.

**Table 8:** Breast diameter in infant girls born to mothers with different vitamin D statuses during pregnancy.

Study Month	Vitamin D Deficient	Vitamin D Insufficient	Vitamin D Sufficient	*p*-Values		
				Deficient vs. Insufficient	Deficient vs. Insufficient	Insufficient vs. Sufficient
1	13 ± 5	12 ± 5	12 ± 5	0.4617	0.4575	1.0000
2	12 ± 5	9 ± 5	8 ± 4	0.0304	0.0017	0.4156
3	12 ± 5	9 ± 4	9 ± 3	0.0175	0.0087	1.0000
4	11 ± 5	8 ± 5	8 ± 4	0.0304	0.0163	1.0000
5	13 ± 5	10 ± 3	9 ± 5	0.0096	0.0046	0.3682
6	12 ± 4	9 ± 4	8 ± 4	0.0075	0.0004	0.3582
8	13 ± 3	10 ± 4	9 ± 3	0.0027	<0.0001	0.2978
10	13 ± 4	10 ± 4	9 ± 5	0.0075	0.0017	0.4175
12	14 ± 3	11 ± 4	8 ± 3	0.0027	0.0028	0.0027
15	13 ± 4	10 ± 4	7 ± 3	0.0075	<0.0001	0.0027
18	14 ± 4	11 ± 4	7 ± 3	0.0028	<0.0001	0.0001

The data are shown as the mean (in mm) ± standard deviation.

**Table 9:** Correlations between the assessed variables.

Correlated Variables		Vitamin D Deficient	Vitamin D Insufficient	Vitamin D Sufficient
Maternal 25OHD	Cumulative vitamin D intake	0.65 [*p* < 0.0001]	0.71 [*p* < 0.0001]	0.76 [*p* < 0.0001]
Estradiol	LH	0.43 [*p* = 0.0004] – 0.65 [*p* < 0.0001]	0.46 [*p* = 0.0002] – 0.64 [*p* < 0.0001]	0.47 [*p* = 0.0001] – 0.64 [*p* < 0.0001]
Ovarian volume	LH	0.28 [*p* = 0.0485] – 0.44 [*p* = 0.0006]	0.31 [*p* = 0.0376] – 0.42 [*p* = 0.0009	0.32 [*p* = 0.0246] – 0.43 [*p* = 0.0008]
Ovarian volume	FSH	0.25 [*p* = 0.0487] – 0.38 [*p* = 0.0019]	0.28 [*p* = 0.0464] – 0.41 [*p* = 0.0011]	0.27 [*p* = 0.0481] – 0.44 [*p* = 0.0006]
Uterine length	Estradiol	0.28 [*p* = 0.0395] – 0.42 [*p* = 0.0008]	0.29 [*p* = 0.0385] – 0.44 [*p* = 0.0006]	0.31 [*p* = 0.0285] – 0.47 [*p* = 0.0002]
Breast diameter	Estradiol	0.34 [*p* = 0.0236] – 0.47 [*p* = 0.0002]	0.36 [*p* = 0.0184] – 0.48 [*p* = 0.0001]	0.35 [*p* = 0.0204] – 0.48 [*p* = 0.0001]
Breast diameter	FSH	0.32 [*p* = 0.0248] – 0.41 [*p* = 0.0012]	0.31 [*p* = 0.0305] – 0.46 [*p* = 0.0002]	0.31 [*p* = 0.0294] – 0.44 [*p* = 0.0005]
Maternal 25OHD	Estradiol	-0.34 [*p* = 0.0214] – -0.46 [*p* = 0.0002]	-0.10 [*p* = 0.4785] – -0.21 [*p* = 0.0841]	-0.12 [*p* = 0.4032] – 0.14 [*p* = 0.2876]
Maternal 25OHD	LH	-0.30 [*p* = 0.0311] – -0.42 [*p* = 0.0006]	-0.08 [*p* = 0.5312] -0.19 [*p* = 0.1121]	-0.14 [*p* = 0.2912] – 0.13 [*p* = 0.3512]
Maternal 25OHD	FSH	-0.43 [*p* = 0.0004] – -0.50 [*p* < 0.0001]	-0.41 [*p* = 0.0010] – -0.48 [*p* = 0.0001]	-0.22 [*p* = 0.0610] – 0.04 [*p* = 0.6425]

The data represent the correlation coefficients (*r* values). Abbreviations: FSH—follicle-stimulating hormone; LH—luteinizing hormone; 25OHD—25-hydroxyvitamin D.

## Data Availability

The original contributions presented in the study are included in the article, further inquiries can be directed to the corresponding author.

## Supplementary Materials

The following supporting information can be downloaded at https://www.mdpi.com/article/10.3390/nu16142362/s1, Table S1: Total daily vitamin D intake in infants girls participating in the study; Table S2: Testosterone concentration in saliva of infant girls born to mothers with different vitamin D status during pregnancy; Table S3: Androstenedione concentration in saliva of infant girls born to mothers with different vitamin D status during pregnancy; Table S4: DHEA-S concentration in saliva of infant girls born to mothers with different vitamin D status during pregnancy; Table S5: Progesterone concentration in saliva of infant girls born to mothers with different vitamin D status during pregnancy; Table S6: 17-hydroxyprogesterone concentration in saliva of infant girls born to mothers with different vitamin D status during pregnancy.

## Abbreviations

BMI—body mass index; DHEA-S—dehydroepiandrosterone sulfate; FSH—follicle-stimulating hormone; LH—luteinizing hormone; LOD—limit of detection; 25OHD—25-hydroxyvitamin D.
