# Supplementary material for: The Course of Minipuberty in Daughters of Women with Low Gestational Vitamin D Status
Source: Nutrients. 2024 Jul 21;16(14):2362. doi: 10.3390/nu16142362 (PMC11279621; doi:10.3390/nu16142362)
Supplement: Supplementary file 1 [file nutrients-16-02362-s001.zip › nutrients-3069795-supplementary.pdf]

Supplementary Table S1. Total daily vitamin D intake in infants girls participating in the study

Study month	Vitamin D deficient	Vitamin D insufficient	Vitamin D sufficient	*p*-values		
				deficient *vs.* insufficient	deficient *vs.* insufficient	insufficient *vs.* sufficient
1	13.1±1.5	13.2±1.7	13.4±1.8	0.8178	0.5010	0.6738
3	13.9 ±1.5	14.2±1.6	14.5±1.9	0.4761	0.1952	0.5300
6	15.3±2.0	14.9±1.6	15.1±1.8	0.4175	0.6956	0.6654
12	20.6±2.7	20.2±2.4	19.8±2.2	0.5644	0.2295	0.5219
18	22.1±2.4	22.8±3.0	22.4±2.7	0.3428	0.6621	0.6051

The data are shown as the mean (in μg) ± standard deviation.

Supplementary Table S2. Testosterone concentration in saliva of infant girls born to mothers with different vitamin D status during pregnancy

Study month	Vitamin D deficient	Vitamin D insufficient	Vitamin D sufficient	*p*-values		
				deficient *vs.* insufficient	deficient *vs.* insufficient	insufficient *vs.* sufficient
1	48±20	51±23	55±30	0.6075	0.3089	0.5823
2	53±25	49±18	51±26	0.5002	0.7603	0.7423
3	50±23	45±21	48±20	0.4042	0.7298	0.5896
4	46±18	50±25	41±22	0.4978	0.3561	0.1618
5	42±17	44±18	39±20	0.6734	0.5479	0.3448

The data are shown as the mean (in pmol/L) ± standard deviation.

Supplementary Table S3. Androstenedione concentration in saliva of infant girls born to mothers with different vitamin D status during pregnancy

Study month	Vitamin D deficient	Vitamin D insufficient	Vitamin D sufficient	*p*-values		
				deficient *vs.* insufficient	deficient *vs.* insufficient	insufficient *vs.* sufficient
1	83±40	74±36	78±35	0.3850	0.6207	0.6207
2	78±25	80±40	84±32	0.8242	0.4378	0.6834
3	88±38	81±37	86±41	0.4921	0.8506	0.6374
4	69±34	74±35	80±38	0.5932	0.2587	0.5455
5	65±29	70±32	73±39	0.5427	0.3876	0.3876

The data are shown as the mean (in pmol/L) ± standard deviation.

Supplementary Table S4. DHEA-S concentration in saliva of infant girls born to mothers with different vitamin D status during pregnancy

Study month	Vitamin D deficient	Vitamin D insufficient	Vitamin D sufficient	*p*-values		
				deficient *vs.* insufficient	deficient *vs.* insufficient	insufficient *vs.* sufficient
1	168±82	150±85	172±74	0.4276	0.3101	0.8488
2	156±76	160±78	164±82	0.8480	0.7092	0.8538
3	146±68	143±70	155±64	0.8725	0.6121	0.5096
4	160±71	168±64	142±71	0.6629	0.3471	0.1601
5	142±64	178±70	160±78	0.0621	0.3494	0.3764
6	170±67	174±80	153±74	0.8412	0.3715	0.3166
8	155±83	128±69	140±71	0.1961	0.4706	0.5279
10	140±70	134±65	146±62	0.7434	0.7355	0.4865
12	135±82	123±80	120±64	0.5853	0.4488	0.8783

The data are shown as the mean (in nmol/L) ± standard deviation.
Abbreviation: DHEA-S - dehydroepiandrosterone sulfate

Supplementary Table S5. Progesterone concentration in saliva of infant girls born to mothers with different vitamin D status during pregnancy

Study month	Vitamin D deficient	Vitamin D insufficient	Vitamin D sufficient	*p*-values		
				deficient *vs.* insufficient	deficient *vs.* insufficient	insufficient *vs.* sufficient
1	71±51	67±42	75±42	0.7526	0.7498	0.4832
2	61±46	53±31	65±39	0.4646	0.7268	0.2131
3	55±39	50±35	70±44	0.6193	0.1827	0.0683
4	80±53	70±41	71±40	0.4385	0.4763	0.9274
5	74±40	72±47	68±39	0.8655	0.5722	0.7322
6	76±48	62±40	65±42	0.2461	0.3655	0.7874
8	55±37	60±35	60±32	0.6091	0.5908	1.0000
10	59±38	53±36	62±29	0.5506	0.7411	0.3110
12	48±35	49±26	53±32	0.9050	0.5792	0.6138

The data are shown as the mean (in pmol/L) ± standard deviation.

Supplementary Table S6. 17-hydroxyprogesterone concentration in saliva of infant girls born to mothers with different vitamin D status during pregnancy

Study month	Vitamin D deficient	Vitamin D insufficient	Vitamin D sufficient	*p*-values		
				deficient *vs.* insufficient	deficient *vs.* insufficient	insufficient *vs.* sufficient
1	53±26	56±38	61±42	0.7329	0.3952	0.6457
2	51±28	52±32	53±35	0.9022	0.8142	0.9125
3	60±32	55±31	53±38	0.5589	0.4591	0.8318
4	56±30	62±35	64±30	0.6540	0.3228	0.8207
5	50±28	56±38	53±40	0.5068	0.7463	0.7808
6	52±23	47±40	51±31	0.5705	0.8915	0.6795
8	49±29	53±29	60±28	0.6112	0.1582	0.3665
10	55±26	58±30	52±23	0.6931	0.6493	0.4079
12	46±20	50±25	47±26	0.5144	0.8724	0.6647

The data are shown as the mean (in pmol/L) ± standard deviation.
